# Effects of a single phosphate-enriched test meal on inflammasome activity and postprandial inflammatory markers in healthy subjects

**DOI:** 10.1007/s00394-023-03306-6

**Published:** 2024-01-04

**Authors:** Anika Nier, Christof Ulrich, Christin Volk, Marie-Charlotte Wolffgang, Corinna Brandsch, Monika Wensch-Dorendorf, Matthias Girndt, Gabriele I. Stangl

**Affiliations:** 1 Institute of Agricultural and Nutritional Sciences, Martin Luther University Halle-Wittenberg, Halle, Germany; 2 Department of Internal Medicine II, Martin Luther University Halle-Wittenberg, Halle, Germany; 3 Competence Cluster of Cardiovascular Health and Nutrition (nutriCARD), Halle-Jena-Leipzig, Germany

**Keywords:** Clinical trial, Phosphorus, Inflammation, Diet, Nutrition, Monocytes

## Abstract

**Purpose:**

The consumption of highly processed food is often associated with a high intake of inorganic phosphate. Hyperphosphatemia is accompanied by an inflammatory status in patients with chronic kidney disease. However, the immune response to high phosphorus intake in healthy individuals is largely unknown. Therefore, the aim of the present study was to evaluate the effect of a single phosphate-enriched meal on inflammasome activity and plasma levels of inflammatory markers.

**Methods:**

The analysis included 28 participants who received a single dose of either 700 mg phosphorus or a placebo with a test meal. At baseline, 4 and 8 h post-meal, plasma interleukin (IL)-6, IL-1β, IL-10, c-reactive protein (CRP), soluble IL-6 receptor (sIL-6R) and glycoprotein 130 (sgp130) levels were determined. At baseline and 4 h post-meal, peripheral blood mononuclear cells were isolated to assess inflammasome activity. Subsequently, the effect of phosphate with or without glucose on *IL-6* and *IL-1β* gene expression and secretion in U937 monocytes was examined.

**Results:**

While both groups showed a marked postprandial increase in IL-6 plasma levels, neither plasma levels of IL-6, IL-1β, CRP, IL-10, sIL-6R, and sgp130 nor inflammasome activity were affected by phosphate compared to placebo. In U937 cells, there was also no effect of phosphate on *IL-6* expression, but the addition of glucose increased it. Phosphate, however, reduced the IL-1β secretion of these cells.

**Conclusion:**

Postprandial inflammatory markers were not affected by dietary phosphate. However, IL-6 plasma levels were markedly increased post-meal, which appears to be a metabolic rather than a pro-inflammatory phenomenon.

**Trial registration number:**

ClinicalTrials.gov, NCT03771924, date of registration: 11th December 2018, retrospectively registered.

## Introduction

Several studies have noted an association between elevated serum phosphate (Pi) levels and an increased risk of cardiovascular disease (CVD) in patients with chronic kidney disease (CKD) [1, 2]. The higher CVD risk in these patients is suggested to be caused primarily by Pi retention, which can affect vascular and cardiac valvular calcification [3–5]. However, assessment of cross-sectional data from CKD patients has suggested an association between elevated serum Pi concentrations and increased levels of interleukin (IL)-6 and C-reactive protein (CRP), indicating that elevated serum Pi levels may also affect inflammation in patients with CKD [6]. The assumption that phosphate may induce inflammation is supported by observations that vascular smooth muscle cells treated with high-phosphate media show increased expression of inflammatory cytokines [7], and adenine-induced CKD rats fed high-phosphate diets display increased serum and tissue concentrations of tumor necrosis factor-α (TNFα) and oxidative stress markers [8]. Interestingly, increased expression of *IL-6* and *IL-1β* in the liver and increased expression of *TNFα* and other pro-inflammatory cytokines in bones have also been found in mice without renal impairment that received excessive phosphate [9]. In vitro data obtained from human aortic smooth muscle cells showed that phosphate provoked not only pro-inflammatory responses but also pro-oxidative conditions, which were accompanied by an increased formation of reactive oxygen species (ROS) and reactive nitrogen species (RNS) [10].

Oxidative stress and the production of mitochondrial ROS might activate inflammasomes [11], molecular complexes that can activate caspase-1 and, in turn, the secretion of IL-1β [12]. IL-1β acts as a primary inflammatory cytokine and induces, among others, the expression of *IL-6* [13]. IL-6 mediates its effects either by binding to the membrane-bound IL-6 receptor of cells (*classic signaling*) or by forming a complex with the soluble form of this receptor (sIL-6R), which subsequently can bind to the membrane-bound glycoprotein 130 (gp130) (*trans-signaling*) [14]. The membrane-bound IL-6 receptor is expressed only in a few cell types, such as hepatocytes or immune cells [14], and mediates the regenerative and anti-inflammatory effects of IL-6 [15], while ubiquitously expressed gp130 [16] can stimulate pro-inflammatory cascades via IL-6 *trans-signaling* [17, 18]. Whether phosphate affects *classic* or *trans-signaling* or both of these signaling pathways is currently unknown.

However, the importance of phosphate in provoking pro-inflammatory and pro-oxidative situations is relevant not only in the context of concomitant diseases in CKD patients but also for healthy subjects because the rising consumption of processed food is accompanied by a high intake of phosphate additives [19]. In the United States, the consumption of highly processed foods is estimated to account for 50–70% of an individual’s total energy intake [20], and approximately half of these foods contain phosphate additives [21]. As a result, the intake of phosphate in Western populations habitually exceeds the quantity of phosphorus recommended by the Institute of Medicine (700 mg/d) [22]. Trautvetter et al. [23], who analyzed data from 149 subjects in Germany, reported a habitual phosphate intake of more than 1300 mg per day. In contrast to phosphate in natural foods, phosphate from inorganic phosphate additives is more effectively absorbed in the intestine [24]. Although circulating Pi is tightly regulated in healthy subjects [25], our previous data show that compared to placebo, a single dose of 700 mg phosphorus administered orally as sodium dihydrogen phosphate (NaH_2_PO_4_, 3.53 g) to healthy subjects results in a significant increase in postprandial plasma Pi levels, which remain elevated over a period of 8 h post-meal (at 480 min: + 13%) [26].

However, the significance of increased plasma Pi levels in healthy subjects in inducing oxidative stress and in turn the stimulation of inflammasome activity, which can trigger the release of IL-1β, IL-6 *trans-signaling* and the secretion of inflammatory markers, is largely unknown. Since the level of circulating phosphate is not permanently elevated in healthy people but only post-meal, we hypothesized that possible effects of phosphate on inflammasome activity and inflammatory markers are primarily seen postprandially, especially given that inflammation markers can increase within a few hours post-meal [27]. To test this hypothesis, the current study investigated the effect of a single phosphate-enriched test meal on inflammasome activity and the plasma levels of inflammatory markers in healthy subjects.

## Materials and methods

### Study population

The participants included in the present study were part of the clinical trial entitled “Postprandial Response of Individuals to Dietary Inorganic Phosphate”. This study focused on the acute effects of dietary phosphate versus placebo on the postprandial plasma levels of Pi, urinary Pi excretion, regulators of mineral homeostasis such as fibroblast growth factor 23 (FGF23) and cardiometabolic risk factors and was published in 2022 [26]. The study was approved by the Ethics Committee of the Medical Faculty at Martin Luther University Halle-Wittenberg and was carried out in accordance with the Declaration of Helsinki. The study was conducted as a double-blind interventional trial with a crossover design at the Department of Internal Medicine II and the Institute of Agricultural and Nutritional Sciences at Martin Luther University Halle-Wittenberg (clinical trials.gov; ID: NCT03771924). For the current analysis, only blood of the first trial sequence was analyzed because the effects of phosphate in comparison to placebo were not different for any of the parameters measured between the two treatment sequences [26]. Thus, the present analysis was treated as a double-blind study with a parallel design.

### Inclusion and exclusion criteria

The inclusion and exclusion criteria as well as the characteristics of the study participants were previously described in detail [26]. In brief, the inclusion criteria were as follows: body mass index (BMI) between 18.5 and 29.9 kg/m^2^; normal kidney function as assessed by plasma creatinine, cystatin C and protein excretion levels and rated by an experienced nephrologist; and plasma 25-hydroxy vitamin D (25(OH)D) levels > 30 nmol/l to ensure that no vitamin D-deficient subjects were included in the study. The exclusion criteria were the presence of allergies or intolerances to the test meal, pregnancy, lactation, chronic disease, medication, smoking, blood donation within the last two months, and participation in other clinical studies.

### Sample size

As already reported by Volk et al. [26], the sample size was calculated (G*Power 3.1.9.2) to receive a significant difference (*p* < 0.05; Power 90%) in the postprandial plasma Pi concentrations between the phosphate and the placebo groups. Based on these specifications, the calculated sample size was 16. To consider possible differences in the effect of phosphate caused by the trial sequence, the sample size was increased to 32. Assuming a drop-out rate of 10%, 36 individuals were included in the study and randomly assigned to either the placebo or phosphate group (also see [26]). During the study, seven participants dropped out for personal reasons, and one person had to be excluded due to missing IL-6 values. Thus, the data for 28 subjects were included in the present analysis.

### Experimental treatment

**Fig. 1:**
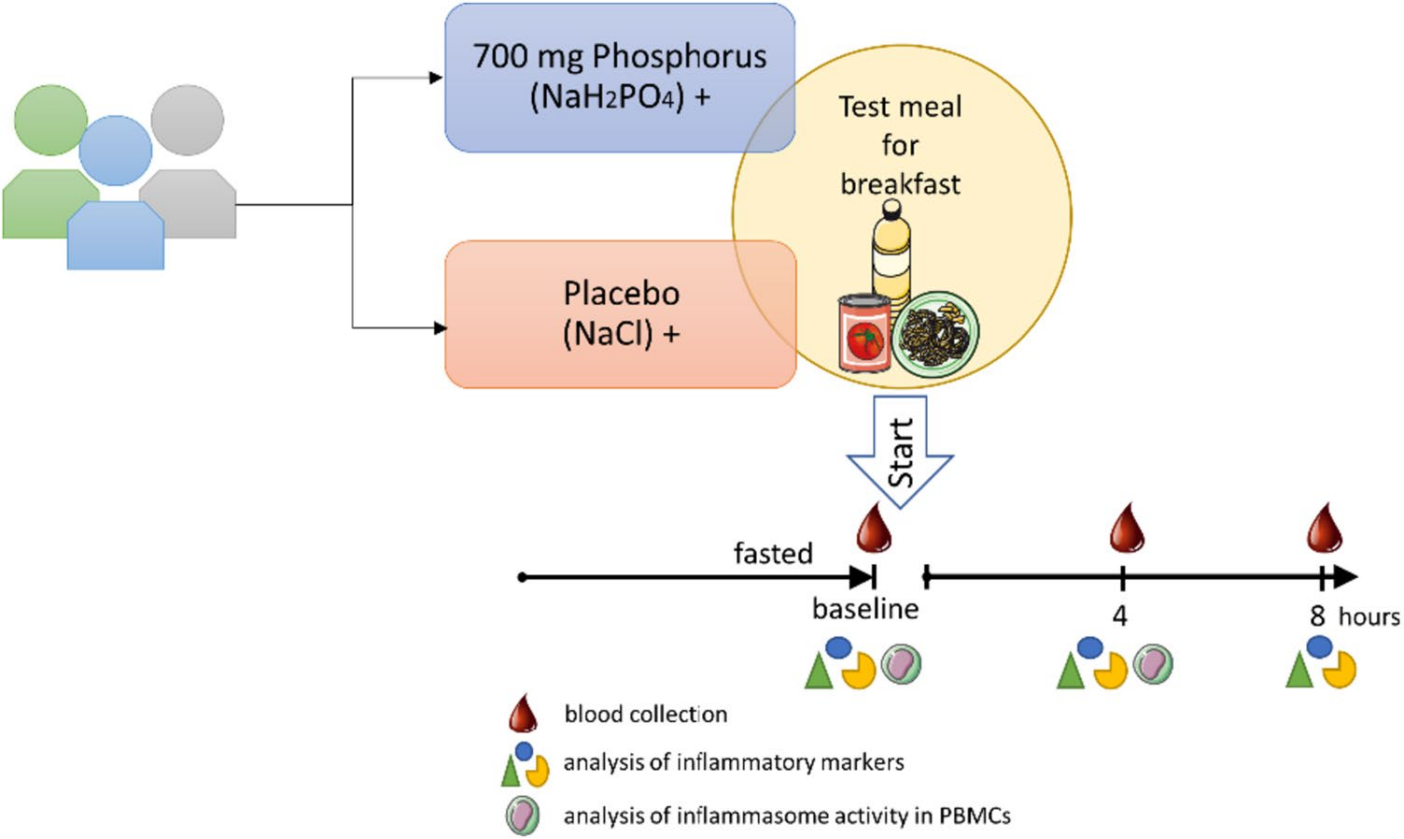
Study design. A test meal together with phosphate or a placebo was served after a 12 h overnight fast. Analyses of plasma were conducted at baseline (− 15 min before the test meal) and 4 and 8 h post-meal, and analyses of isolated peripheral blood mononuclear cells (PBMCs) were conducted at baseline and 4 h post-meal

The study design was previously described in detail [26]. To ascertain whether the inclusion criteria were met, BMI, kidney function, and 25(OH)D levels were assessed 2–3 weeks prior to the beginning of the study. Therefore, all subjects had to complete a questionnaire to assess demographic and anthropometric data, and blood and urine samples were collected. Participants enrolled in the study were randomly assigned to two groups and received a single dose of either 700 mg phosphorus (3.53 g NaH_2_PO_4_) or a placebo containing NaCl (to keep sodium intake the same in both groups) together with a test meal (150 g boiled pasta, 180 g tomato sauce, 20 g corn oil) (Fig. 1). NaH_2_PO_4_ and NaCl were administered as capsules of identical appearance. The intervention started in the morning after a 12 h overnight fast. A blood sample taken 15 min before the intervention was used to analyze baseline levels.

The figure was created with images adapted and modified from Servier Medical Art by Servier licensed under a Creative Commons Attribution 3.0 Unported License (https://smart.servier.com/; https://creativecommons.org/licenses/by/3.0/legalcode).

### Anthropometric evaluation, blood sampling and laboratory procedures

BMI, blood pressure, glucose and lipids were assessed as described recently [26]. In brief, weight and height to calculate BMI were self-reported by participants. Blood pressure was assessed on the dominant arm in triplicate with a 1-min interval using automated portable upper-arm blood pressure monitors, and plasma concentrations of glucose and lipids were assessed at the central laboratory of the university hospital of Martin Luther University Halle-Wittenberg. To assess whether BMI, blood pressure, and blood lipids were in the normal or pathological ranges, the classification of the World Health Organization (WHO) was used for BMI, the European Society of Hypertension (ESH) guidelines for blood pressure [28], and “*The Task Force for the Management of Dyslipidaemias of the European Society of Cardiology (ESC) and European Atherosclerosis Society (EAS)*” guidelines for lipids [29].

Venous blood samples collected at baseline and 4 and 8 h post-meal were used to analyze the plasma levels of IL-6, IL-1β, CRP, IL-10, sIL-6R and sgp130. Peripheral blood mononuclear cells (PBMCs) for inflammasome activity measurements were isolated from blood obtained at baseline and 4 h postprandial, the time point with the highest Pi levels [26]. The 8 h postprandial levels were analyzed to consider the well-known time-delayed release of pro-inflammatory cytokines into the plasma [30, 31]. Plasma levels of IL-6, high sensitivity IL-1β (hsIL-1β), sIL-6R (all from IBL international GmbH, Tecan group), IL-10 (R&D Systems Inc.), soluble gp130 (sgp130) (RayBiotech Life, Inc.) and CRP (DRG Diagnostics GmbH) were determined using commercially available ELISA kits according to the manufacturer’s instructions.

### Isolation of peripheral blood mononuclear cells and analysis of inflammasome complexes

To test whether the single oral intake of the phosphate-rich meal leads to elevated mitochondrial stress and consequently to the activation of inflammasome complexes, mitochondrial ROS, caspase-1 activity and pyroptosis were assessed in PBMCs at baseline and 4 h post-meal. The isolation of PBMCs and the analysis of inflammasome complexes were performed as described previously [32]. In brief, PBMCs were isolated from whole-blood samples by ficollization (GE Healthcare, Solingen, Germany). The viability of PBMCs was tested with 7-aminoactinomycin D (7-AAD) staining (Thermo Fisher Scientific, Darmstadt, Germany). The viability of PBMCs was > 99% for all participants. Representative of nucleotide-binding oligomerization domain (NOD)-like receptor (NLR) family pyrin domain containing 3 (NLRP3) inflammasome activation, the caspase-1 activity was determined under basal and stimulated conditions. For stimulation of cells, lipopolysaccharide (LPS from *E. coli* 0111:B4; 1 µg/ml, Sigma‒Aldrich, Steinheim, Germany) and nigericin (5 µg/ml, Sigma‒Aldrich) were used to induce a high caspase-1 response. In contrast to LPS, nigericin (acting as a K^+^ ionophore) was applied for the last 15 min of the incubation period. In brief, 0.25 × 10^6^ PBMCs were incubated at 37 °C in a 5% CO_2_ atmosphere. After an incubation period of 4 h, caspase-1 was detected by flow cytometry (MACS Quant analyzer, Miltenyi Biotec, Bergisch-Gladbach, Germany) with the FAM (fluorescent carboxyfluorescein)-YVAD (Tyr-Val-Ala-Asp)-FLICA (fluorescent-labeled inhibitors of caspases) inhibitor probe (Bio-Rad, Feldkirchen, Germany). One hour before ending the regular incubation period, the cells were pelleted and resuspended in sterile PBS/0.5% human serum albumin (HAS) containing the FAM-YVAD-FLICA inhibitor probe. The incubation was continued for 1 h. Samples without caspase-1 inhibitor were used as negative controls. Cells were counterstained using labeled anti-monocyte- (cluster of differentiation 14; CD14) and anti-lymphocyte- (cluster of differentiation 3; CD3) specific antibodies (Thermo Fisher Scientific). The caspase-1 activity of the cells was expressed as the mean fluorescence intensity (MFI). Inflammatory cell death (pyroptosis) was measured by analysis of 7-AAD and caspase-1 double-positivity (%).

### Mitochondrial oxidative stress

Mitochondrial ROS were determined by flow cytometry with the fluorogenic indicator probe MitoSOX™ (Thermo Fisher). Therefore, 0.25 × 10^6^ PBMCs were cultivated for a total of 2 h. For the last 15 min of the incubation period, cells were labeled with 2 µM MitoSOX™. Cells without MitoSox™ staining served as negative controls. The data are expressed as MFI.

### Cell culture, RNA isolation and real-time reverse-transcription polymerase chain reaction

To investigate the effects of phosphate on *IL-6* and *IL-1β* expression and to dissect the effects of phosphate and nutrients, particularly glucose, on the observed postprandial increase in circulating IL-6, cell culture studies using U937 monocytes (Leibniz-Institut DSMZ GmbH, Braunschweig, Germany) were carried out. Glucose was chosen for cell culture experiments to mimic the postprandial state because the test meal was rich in carbohydrates (116 g) but low in fat (24 g) [33]. For this purpose, cells were cultured in Roswell Park Memorial Institute (RPMI) 1640 media supplemented with 10% fetal bovine serum (FBS) and 0.5% gentamicin at 37 °C and 5% CO_2_. For the experiments, 1 × 10^6^ cells were seeded per well of 24-well plates. After a rest of 2 h, cells were treated with either glucose-free RPMI 1640 media with 10% FBS alone (negative control) or with LPS (1 µg/ml, positive control) or with 10, 25, or 50 mM glucose, with or without the addition of 10 mM phosphate (ratio: Na_2_HPO_4_/NaH_2_PO_4_, 4:1). For analysis of mRNA expression, cells were treated with the corresponding substances for 2 h. For analysis of secreted ILs, cells were treated for 24 h.

Following the 2 h treatment, cells were harvested, and total RNA was isolated using Tri-Reagent (Sigma‒Aldrich) according to the manufacturer’s instructions. Moloney Murine Leukemia Virus Reverse Transcriptase (M-MLV reverse transcriptase) (Promega Corp.) was used for cDNA synthesis. The relative mRNA abundance of *IL-6* and *IL-1β* was analyzed using real-time reverse-transcription polymerase chain reaction (real-time RT‒PCR). GoTaq Flexi DNA Polymerase (Promega Corp.) was used to amplify a total of 1 µl cDNA template with the Rotorgene 6000 system (Corbett Research Ltd.). The comparative threshold cycle (C_T_) method [34] was used to determine the relative abundance of target gene mRNA normalized to the endogenous reference gene glyceraldehyde-3-phosphate dehydrogenase (GAPDH). The primer sequences were as follows (GAPDH: forward: GACCACAGTCCATGCCATCAC, reverse: TCCACCACCCTGTTGCTGTAG, NM_001357943.2; IL-6: forward: GCAGAAAAAGGCAAAGAATC, reverse: CTACATTTGCCGAAGAGC, NM_001371096.1; IL-1β: forward: CTAAACAGATGAAGTGCTCC, reverse: GGTCATTCTCCTGGAAGG, NM_000576.3). Following the 24 h incubation, the cell culture supernatant was collected for quantification of secreted IL-6 (R&D Systems Inc.) and IL-1β protein (IBL international GmbH, Tecan group) using commercially available ELISA kits according to the manufacturer’s protocol.

### Statistical analysis

All data were analyzed using the Statistical Analysis System (SAS) 9.4 software package (SAS Institute Inc., Cary, NC, USA). The data were tested for normal distribution or, in the absence of normal distribution, for lognormal distribution (plasma IL-6, CRP, *IL-6* mRNA expression and secretion in U937 monocytes). Baseline characteristics were analyzed using a *t test*. For the analysis of cytokine concentrations, pyroptosis and cell culture studies, the *mixed-model procedure (PROC MIX)* was used. The effects of phosphate treatment, time of intervention and their interaction were considered fixed, and participants were considered random. Due to the differences between the groups at baseline, the baseline values were also included in the model as covariates for plasma sIL-6R and IL-10. For the cell culture studies, glucose treatment, phosphate treatment and their interaction were considered fixed, and the repetition was considered random in the model. Due to missing normally distributed data, mitochondrial ROS and caspase activity were analyzed using the *Wilcoxon rank-sum test* with the SAS procedure *npar1way* as a nonparametric alternative to the *two-sample t test*. The plasma parameter data for 4 h post-meal for one subject were missing. The results are presented as total numbers, boxplots (using the Tukey method) and means ± standard deviations (SD). Differences with *p* values < 0.05 were considered significant. GraphPad Prism 9 (San Diego, CA, USA) was used to create figures.

## Results

### Characteristics of study participants

The participants enrolled in the study were aged between 18 and 35 years (Table 1). At baseline, the two groups did not differ in BMI, blood pressure, blood glucose or lipid levels (Table 1). The majority (~ 80%) of all subjects had a BMI within the normal range of 19–25 kg/m^2^, while five individuals had a BMI between 25 and 28 kg/m^2^ (three in the placebo group and two in the phosphate group). Fasting blood concentrations of glucose and triglycerides were within normal ranges. According to ESH [28] and ESC/EAS [29] guidelines, one person in the placebo group had an elevated systolic blood pressure (148 mmHg), and one person in this group was hypercholesterolemic (5.8 mmol/l). In addition, six participants (three in each group) had blood LDL cholesterol concentrations above the cutoff value for individuals at low cardiovascular risk (> 3 mmol/l) [29].

**Table 1:** Characteristics of the study participants at baseline

	Placebo group	Phosphate group
Number of participants	14	14
Sex (male/female)	4/10	3/11
Age (years)	22.2 ± 4.6	22.2 ± 2.6
BMI (kg/m^2^)	22.1 ± 3.2	22.5 ± 1.9
SBP (mmHg)	117.8 ± 11.9	111.9 ± 10.3
DBP (mmHg)	72.1 ± 6.2	71.1 ± 5.1
Plasma glucose (mmol/l)	4.8 ± 0.3	4.7 ± 0.3
Plasma triglycerides (mmol/l)	0.9 ± 0.3	0.8 ± 0.4
Plasma total cholesterol (mmol/l)	4.0 ± 0.8	3.7 ± 0.6
HDL cholesterol (mmol/l)	1.6 ± 0.4	1.4 ± 0.2
LDL cholesterol (mmol/l)	2.5 ± 0.6	2.4 ± 0.5

*BMI* body mass index, *DPB* diastolic blood pressure, *HDL* high-density lipoprotein, *LDL* low-density lipoprotein, *SPB* systolic blood pressure; the data are shown as the mean ± SD

### Effect of phosphate intake on postprandial plasma levels of inflammatory markers

**Fig. 2:**
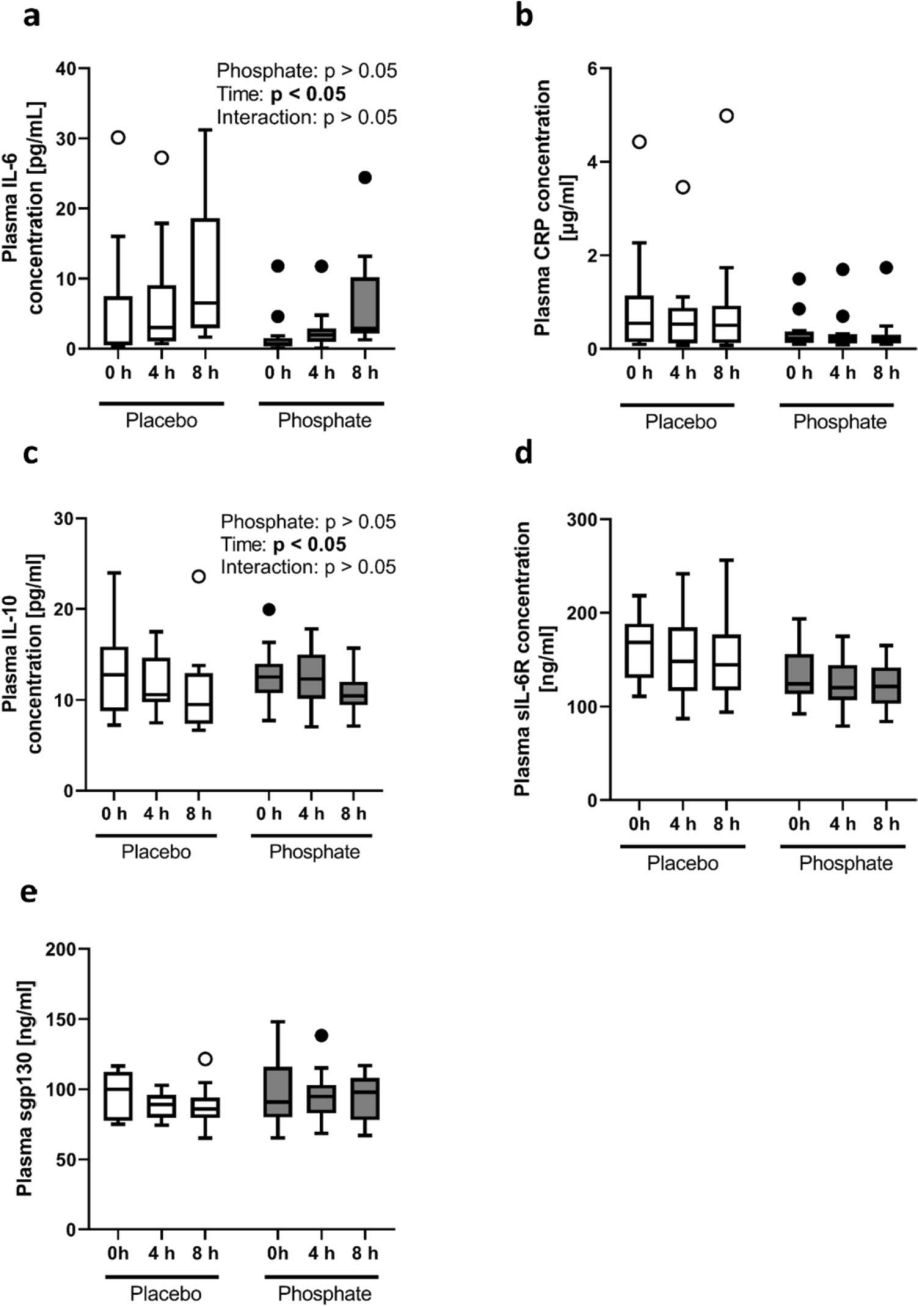
Plasma concentrations of **a** interleukin-6 (IL-6), **b** C-reactive protein (CRP), **c** interleukin-10 (IL-10), **d** soluble IL-6 receptor (sIL-6R) and **e** soluble glycoprotein 130 (sgp130) at baseline (0 h) and 4 h and 8 h after intake of the test meal with either a placebo (Placebo) (white bars) or 700 mg phosphorus (Phosphate) (gray bars). *N* = 14 per group; one value is missing in the placebo group at 4 h post-meal. The data are presented as boxplots [using the Tukey method]

The most important findings were that the postprandial concentrations of IL-6, CRP, IL-10, sIL-6R, and sgp130 did not differ between the phosphate and placebo groups (Fig. 2). Circulating IL-1β was not measurable at baseline or postprandial in the majority of subjects. Interestingly, both groups showed a significant postprandial increase in IL-6 that reached values above the upper limit of normal ranges (> 10 pg/ml) in 40% of the individuals 8 h post-meal (Fig. 2a).

### Effect of phosphate intake on the inflammasome activity of peripheral blood mononuclear cells

**Fig. 3:**
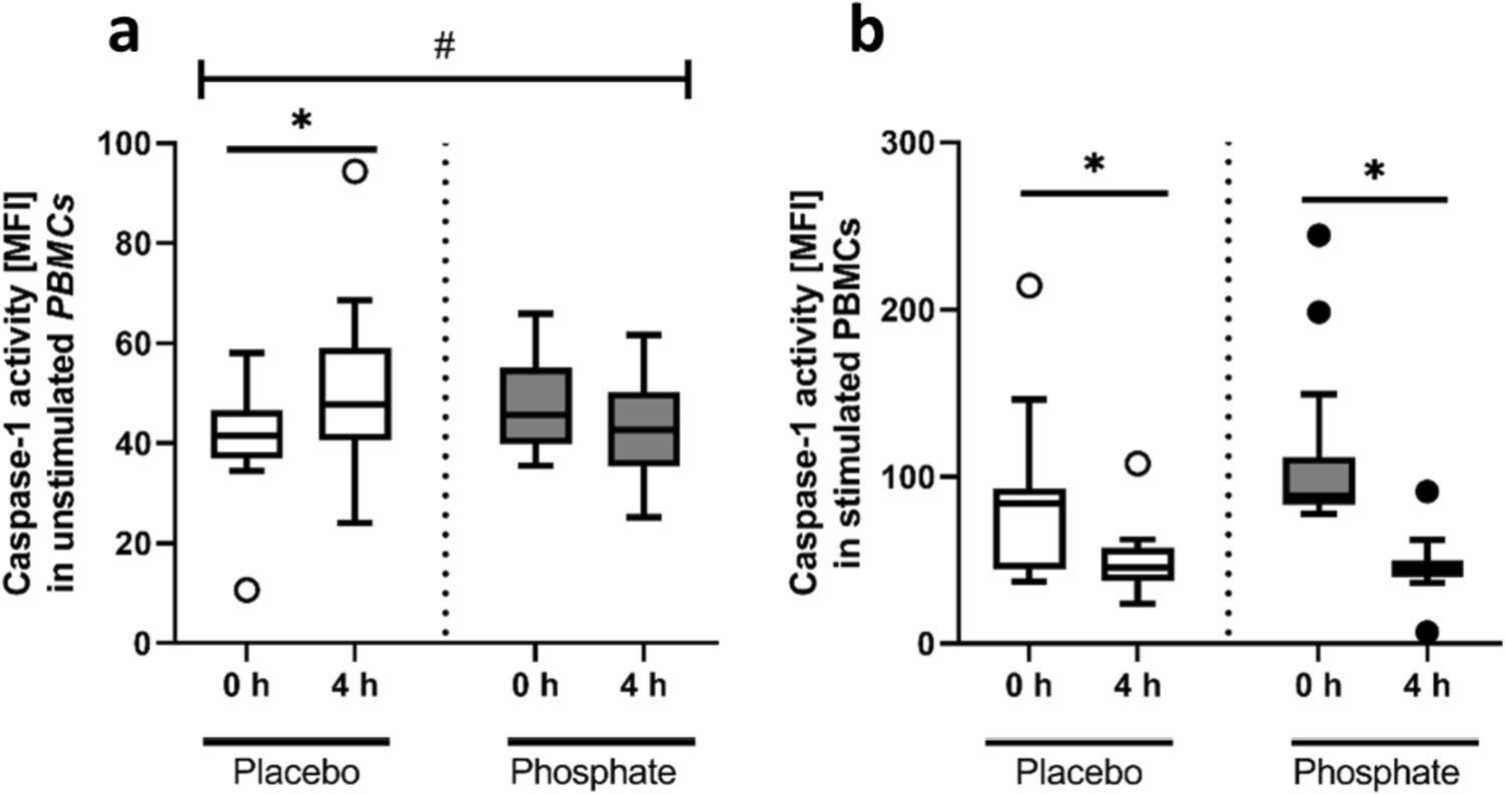
Caspase-1 activity expressed as the mean fluorescence intensity [MFI] in **a** unstimulated and **b** lipopolysaccharide (LPS)-stimulated peripheral blood mononuclear cells (PBMCs) of participants at baseline (0 h) and 4 h after intake of the test meal with either a placebo (Placebo) (white bars) or 700 mg phosphorus (Phosphate) (gray bars). The data are presented as boxplots [using the Tukey method]. *p < 0.05 compared to baseline within a group, ^#^*p* < 0.05 comparing changes between the groups

In unstimulated PBMCs (without added LPS) isolated 4 h post-meal, the quantity of mitochondrial ROS and pyroptosis did not differ between the two groups (Table 2), and there were no post-meal changes (4 h post-meal compared to baseline) in mitochondrial ROS (Table 2). Compared to baseline, PBMCs of both groups were characterized by a small decline in postprandial pyroptosis (Table 2). Interestingly, the postprandial activity of caspase-1 in unstimulated cells remained unchanged in the phosphate group, while it increased in the placebo group (25%; *p* < 0.05; Fig. 3a). In contrast, PBMCs stimulated with LPS showed a decline in caspase-1 activity 4 h post-meal compared to baseline (− 42% for placebo; − 59% for phosphate; both: *p* < 0.05), which was, by trend, more pronounced in stimulated PBMCs of the phosphate group than in those of the placebo group (*p* = 0.08; Fig. 3b).

**Table 2:** Mitochondrial ROS and pyroptosis in unstimulated PBMCs of participants receiving the test meal with either a placebo or 700 mg phosphorus at baseline and 4 h post-meal

	Placebo group		Phosphate group	
	Baseline	4 h	Baseline	4 h
Number of participants	14	14	14	14
Mitochondrial ROS (MFI)	14.7 ± 19.5	10.0 ± 16.6	15.2 ± 16.7	10.6 ± 14.8
Pyroptosis (AAD + caspase-1 + frequency, %)*	7.4 ± 2.9	5.0 ± 3.6	6.3 ± 3.0	4.6 ± 2.7

*MFI* mean fluorescence intensity, *PBMC* peripheral blood mononuclear cells, *ROS* reactive oxygen species

*Effect of phosphate: not significant, effect of time: *p* < 0.05, interaction: not significant. The data are presented as the mean ± SD

### Effects of phosphate and glucose on IL-6 and IL-1β expression and secretion in vitro

**Fig. 4:**
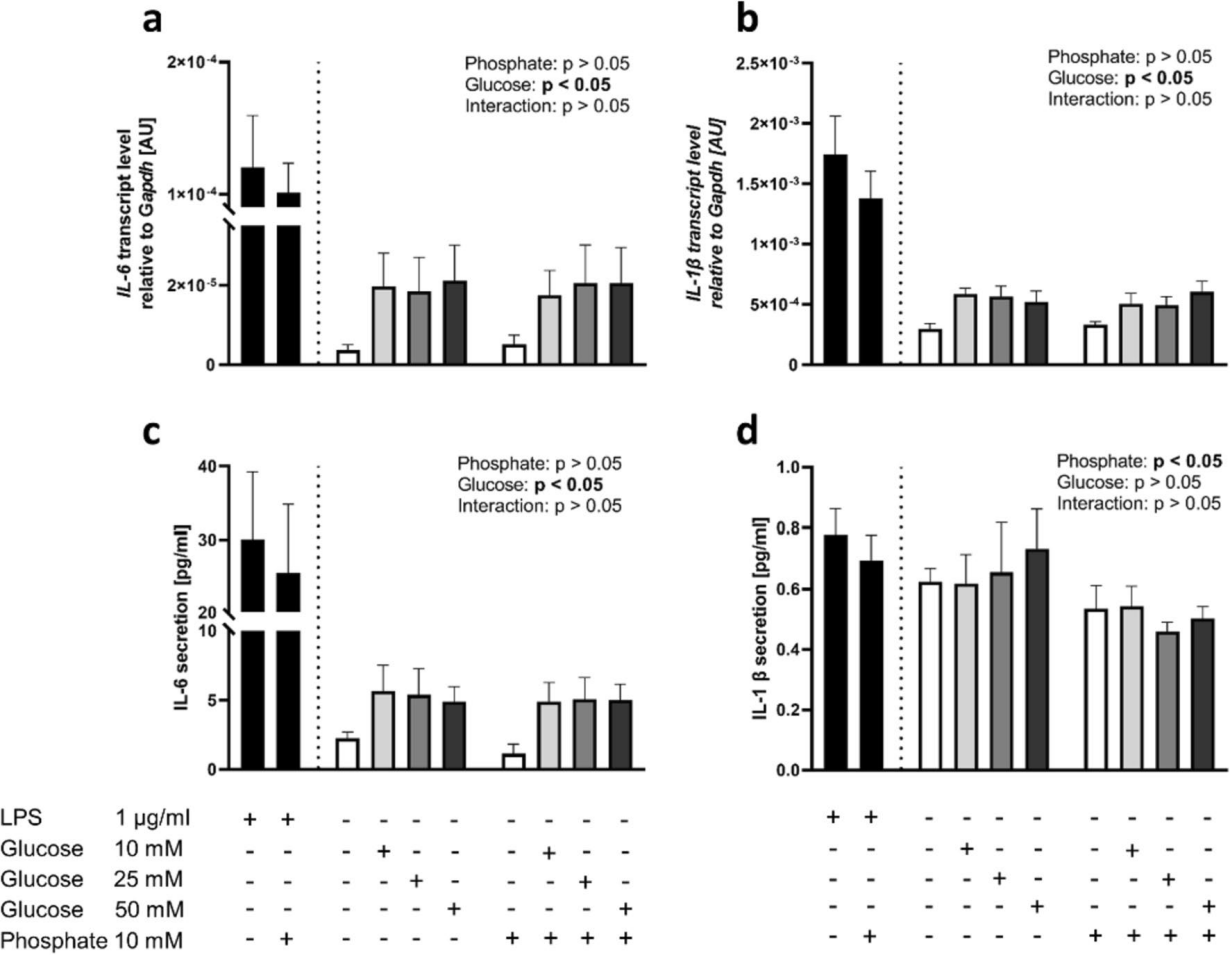
Transcript levels of **a** *interleukin-6 (IL-6)* and **b** *interleukin-1 β (IL-1β)* in U937 cells after 2 h treatment and concentrations of **c** IL-6 and (d) IL-1β in supernatant after 24 h incubation with medium alone (white bars), with added lipopolysaccharide (LPS) (black bars), and with addition (+) of different glucose concentrations [10 mM, 25 mM, 50 mM] (gray bars), with (+) or without (–) addition of phosphate [10 mM]. The data are presented as the mean ± SEM. The LPS data are shown as a positive control and were not included in statistical analyses

Treatment of U937 cells with 10 mM phosphate did not affect the mRNA abundance of *IL-6* or *IL-1β* or the IL-6 secretion of these cells (Fig. 4a–c). More importantly, cells treated with phosphate showed lower secretion of IL-1β than cells treated without phosphate (*p* < 0.05, Fig. 4d), indicating that phosphate had no pro-inflammatory effect. However, the treatment of cells with glucose resulted in a significant increase in the mRNA abundance of *IL-6* and *IL-1β* and IL-6 secretion (*p* < 0.05, Fig. 4a–c) but not in IL-1β secretion, suggesting that the postprandial IL-6 increase observed in the study subjects was the result of carbohydrate intake as part of the test meal.

## Discussion

The current study investigated the acute effects of a single phosphate-enriched meal on inflammation, assessed by pro-inflammatory cytokines and soluble cytokine receptors in plasma as well as inflammasome activity in PBMCs. The data showed that phosphate in comparison to placebo altered neither the levels of pro-inflammatory cytokines nor inflammasome activity. These findings were corroborated by the observed postprandial decline in caspase activity in isolated PBMCs of the phosphate group and results from cell culture studies that showed no pro-inflammatory response after phosphate treatment. Thus, the current results, which are not indicative of any inflammatory stress following phosphate intake, are in contrast to data linking phosphate to inflammation in CKD patients with hyperphosphatemia and animal models incorporating excessive Pi feeding [6, 8].

Data on the underlying mechanisms of Pi in inflammation come primarily from animal and cell studies and suggest increased ROS formation following Pi treatment [8, 10]. Wei et al. [35] showed that high Pi levels can induce the transcriptional activity of NF-E2-related factor 2 (NRF 2) and promote its translocation into the nucleus in vascular smooth muscle cells (VSMCs). These authors concluded that the activation of NRF 2, which results in increased expression of anti-oxidant and anti-inflammatory genes, is a mechanism that counteracts the oxidative stress induced by excessive Pi [35]. Conversely, the current human study did not indicate any differences in mitochondrial ROS between the phosphate and placebo groups. However, notably, we did not use an excessive dose of phosphate but rather administered an acute dose of the entire recommended daily amount [22], which resulted in moderately higher plasma Pi levels and a higher incremental area under the curve (iAUC) of phosphate 8 h post-meal when compared to placebo (mean difference in iAUC phosphate: 139 mmol/l × 480 min) [26]. Therefore, we conclude that a single oral dose of moderate amounts of phosphate in healthy subjects leading to a slight but significant increase in Pi plasma levels might not promote mitochondrial oxidative stress or inflammation in healthy subjects.

The absence of an inflammatory response in phosphate-treated healthy individuals in contrast to patients with CKD [6] might result from the fact that CKD patients, at least in the later stages of the disease, suffer from permanent highly elevated serum Pi levels [36]. Hyperphosphatemia is usually accompanied by the secretion of FGF23 [37], a phosphaturic hormone that is not only inducible by acute and chronic inflammatory factors [38] but also associated with inflammation, as shown in several clinical studies [39–41]. Additionally, in vitro and animal studies have shown that FGF23 stimulates the formation and release of cellular IL-6 and TNF α [42], and genome-wide analysis has revealed that FGF23 is a regulator of pro-inflammatory genes such as *TNF α*, *IL-2*, *IL-4* and *IL-6* in different cell types, such as mast cells or T cells [43]. Importantly, the phosphate-treated subjects in the current study did not show any increase in postprandial FGF23 levels [26], which may have been due to the short study period or the low calcium intake with the meal. Thus, it is tempting to speculate that the noninflammatory effect of phosphate in the current study could result from the absence of an increase in FGF23 levels. Since long-term studies have found that high phosphate intake is associated with elevated FGF23 plasma levels in healthy subjects [44, 45], it would be interesting to investigate inflammation biomarkers in subjects treated with phosphate for several weeks.

As expected, the absent effect of phosphate on inflammasome activity in the current study was accompanied by an absent increase in inflammatory markers such as IL-6, IL-1β, sIL-6R or sgp130. These findings, together with the data obtained from the cell culture study, which are not indicative of any inflammatory effect of phosphate, suggest that an acute oral dose of phosphate did not induce inflammation. Interestingly, the pronounced postprandial increase in IL-6 without any difference between the two groups was not caused by phosphate. Although the IL-6 levels remained elevated for at least 8 h postprandial and were even in the pathological range in 40% of the subjects, this phenomenon is probably not the result of an inflammatory process because other inflammatory markers remained within normal ranges. It can be speculated that the rise in plasma IL-6 levels was a metabolic rather than an inflammatory response. A postprandial increase in IL-6 was also found in other studies with healthy subjects [27, 33, 46]. Recent data suggest that IL-6, which is also released during exercise, is necessary to maintain energy homeostasis [47]. In addition to nutrient intake, IL-6 is known to display a diurnal secretory pattern in humans with low levels of IL-6 in the morning hours and high levels at night [48]. It can, therefore, not be ruled out that the diurnal pattern of IL-6 was, at least in part, responsible for the observed increase in IL-6.

### Strengths and limitations of this study

This study investigated a series of classical pro-inflammatory cytokines and CRP, inflammasome activity and soluble cytokine receptors. Additionally, in contrast to other intervention studies on phosphate, the current trial included a relatively high number of participants, and the effect of phosphate on pro-inflammatory cytokines was also tested in a separate cell culture study.

However, the present study also had some limitations. The calculated sample size was not designed to detect possible differences in inflammatory markers. The study was rather descriptive, and the underlying mechanisms for the postprandial IL-6 increase could not fully be explained. Additionally, the current study investigated only the acute phosphate effects. Thus, any conclusions about the long-term effects of phosphate on inflammation and higher phosphate doses are not possible.

## Conclusion

The data from the current study did not show an inflammatory response of acute phosphate intake because neither pro-inflammatory cytokines nor inflammasome activity in PBMCs were affected in subjects who consumed a test meal with a phosphate additive. However, it cannot be excluded that long-term intake of diets rich in phosphate may lead to inflammatory conditions, e.g., via FGF23. The observed increase in postprandial IL-6 is induced by other nutrients, such as glucose, rather than by phosphate.

## Data Availability

The datasets generated and analyzed during the current study are not publicly available but are available from the corresponding author upon reasonable request.
